# Effect of Empagliflozin and Dapagliflozin on Ambulatory Arterial Stiffness in Patients with Type 2 Diabetes Mellitus and Cardiovascular Co-Morbidities: A Prospective, Observational Study

**DOI:** 10.3390/medicina58091167

**Published:** 2022-08-27

**Authors:** Dimitrios Patoulias, Christodoulos Papadopoulos, Ioanna Zografou, Alexandra Katsimardou, Asterios Karagiannis, Michael Doumas

**Affiliations:** 1 Second Propaedeutic Department of Internal Medicine, General Hospital “Hippokration”, Aristotle University of Thessaloniki, 54642 Thessaloniki, Greece; 2 Third Department of Cardiology, General Hospital “Hippokration”, Aristotle University of Thessaloniki, 54124 Thessaloniki, Greece

**Keywords:** SGLT-2 inhibitor, empagliflozin, dapagliflozin, arterial stiffness, pulse wave velocity, cardiovascular disease

## Abstract

*Background and Objectives*: Individuals with type 2 diabetes mellitus (T2DM) have an increased risk of cardiovascular disease. Arterial stiffness is an independent prognostic marker for cardiovascular disease development. We aimed at determining the effect of two different sodium-glucose co-transporter-2 (SGLT-2) inhibitors on ambulatory arterial stiffness in individuals with T2DM. *Materials and Methods*: In this single-center, single-arm, prospective study performed from January 2020 to August 2021, we planned to enroll adult subjects with T2DM and stable antidiabetic and antihypertensive treatment, assigned either to empagliflozin or dapagliflozin for 6 months. All eligible subjects underwent ambulatory blood pressure monitoring. We set as the primary efficacy outcome the change in ambulatory pulse wave velocity (PWV) from baseline to week 24. *Results*: We finally enrolled 46 diabetic subjects, with a mean age of 62.89 (8.53) years and mean T2DM duration of 9.72 (6.37) years. Thirty patients received dapagliflozin, while sixteen patients received empagliflozin. Due to COVID-19 pandemic restrictive measures during the study, the mean follow-up period extended from 6 months to 9.98 (3.27) months. Regarding the prespecified primary efficacy outcome, we found that the SGLT-2 inhibitor treatment did not have a significant effect on PWV (*p* = 0.65). Prior history of cardiovascular disease did not significantly affect the observed effects. Other indices of arterial stiffness, such as augmentation index and central pulse pressure, were not significantly affected, neither by empagliflozin nor by dapagliflozin. *Conclusions*: SGLT-2 inhibitor treatment with empagliflozin or dapagliflozin in subjects with T2DM failed to improve ambulatory PWV over a mean follow-up of 10 months. Registration number: ISRCTN88851713.

## 1. Introduction

Individuals with type 2 diabetes mellitus (T2DM) have an increased risk of cardiovascular disease (CVD) development during the disease course [1,2]. Up to one-third of diabetic subjects manifest some form of CVD, while it accounts for death in 9.9% of them [1]. Additionally, subjects with T2DM have a significantly increased risk for cardiovascular death by 14% compared to non-diabetic controls [3]. Besides the undoubted impact on patients’ quality of life, CVD has an important socioeconomic impact on national healthcare systems [4].

Hyperglycemia and insulin resistance contribute to enhanced oxidative stress, low-grade chronic inflammation, endothelial dysfunction, increase in advance glycation end-products and protein kinase C overactivation, and thus to increased atherogenesis and atherothrombosis [5]. Other important mechanisms for enhanced atherogenesis among patients with T2DM include platelets’ dysfunction, hypercoagulability, elevated blood viscosity, elevated fibrinogen levels and promotion of a more atherogenic phenotype of vascular smooth muscle cells, resulting in a more complex and slightly different pathogenesis of atherosclerosis, compared to non-diabetic individuals [6]. Due to its complex pathogenesis, T2DM represents an independent risk factor for vascular re-stenosis and in-stent re-stenosis, after endovascular treatment for coronary artery disease, carotid artery disease or peripheral artery disease (i.e., secondary prevention) [6], associated with higher morbidity and mortality, development of frailty and subsequently worse quality of life and susceptibility to other co-morbidities [7].

Thus, there is an absolute need for new, useful and easily measured prognostic markers, which can be utilized for the primary and secondary prevention of major adverse cardiovascular events (MACEs) and cardiovascular death in those patients [8].

Arterial stiffness constitutes a prognostic marker of CVD, whereas pulse wave velocity (PWV) is the “gold-standard” for its quantification in daily clinical routine. Previous meta-analyses have shown the value of PWV for the prediction of future MACEs, cardiovascular and all-cause mortality, with a cut-off value of 10 m/s [9,10,11]. However, only a few dedicated observational studies have addressed the prognostic impact of PWV on cardiovascular morbidity and mortality in subjects with T2DM, highlighting its role in the field of primary and secondary prevention [12,13,14]. All these studies evaluated office PWV, with the use of tonometry. On the other hand, there are only limited data regarding the prognostic role of ambulatory PWV in CVD prediction, demonstrating that ambulatory and office PWV are equivalent [15]. Despite being scarce, recent evidence has shown sufficient agreement between office and ambulatory PWV, although proper validation studies are required [16,17].

Sodium-glucose co-transporter-2 (SGLT-2) inhibitors have notable cardiovascular efficacy, as shown in the relevant cardiovascular outcome trials [18]. Thus, they are now indicated for individuals with T2DM and CVD, or at a high/very high risk of CVD [19]. In addition, they appear as efficacious treatment options in patients with heart failure (HF) or CKD, even without underlying T2DM [20,21,22,23,24]. Of note, underlying mechanisms mediating their favorable cardiovascular and renal effects are hypothesized but have not been definitely confirmed [25]. An improvement in arterial stiffness seems to be a reasonable underlying pathophysiologic mechanism: SGLT-2 inhibitors improve glycemia, promote osmotic diuresis leading to reduction in blood pressure, improve endothelial function, suppress oxidative stress and exert anti-inflammatory effects [26], all being mechanisms implicated into increased arterial stiffness.

Therefore, we attempted to determine whether two different SGLT-2 inhibitors with established cardiovascular efficacy exert any effect on ambulatory PWV and other indices of arterial stiffness, by conducting a pilot, observational study in a real-world setting.

## 2. Methods

### 2.1. Study Design and Protocol

This is a single-center, prospective, observational study, conducted in Greece, from January 2020 to August 2021. The study was registered at the International Standard Registered Clinical/soCial sTudy Number (ISRCTN) registry (ISRCTN88851713).

The study design and protocol have already been described in detail in a previous publication by our research team [27].

### 2.2. Intervention

Enrolled patients were assigned dapagliflozin or empagliflozin, as described previously [27].

### 2.3. Outcomes of Interest

We set as the primary efficacy outcome the change in ambulatory pulse wave velocity (PWV) from baseline to week 24. Secondary efficacy outcomes of interest included: augmentation index (Aix); pulse pressure (PP); 24 h systolic and diastolic blood pressure (24-h SBP and DBP, respectively); central systolic and diastolic blood pressure (cSBP and cDBP, respectively); 24 h heart rate (24-h HR). We also assessed several other markers of specific interest, including change in glycemic control [HbA1c and fasting plasma glucose (FPG)]; body weight and body mass index; office systolic and diastolic blood pressure; office heart rate; renal function [serum creatinine and estimated glomerular filtration rate as measured by CKD-EPI (Chronic Kidney Disease Epidemiology Collaboration) equation]; 24 h urinary protein excretion and urine microalbumin in spot urine collection; hematocrit and hemoglobin; lipid profile parameters [serum total cholesterol (TC), low-density lipoprotein cholesterol (LDL-C), high-density lipoprotein cholesterol (HDL-C) and triglyceride (TRG)]; C-reactive protein (CRP) levels, from baseline to week 24.

We also evaluated safety outcomes of interest throughout the study, including any and severe hypoglycemia; acute hyperglycemic crisis, mainly diabetic ketoacidosis; urinary and genital tract infection; lower limb amputation; orthostatic hypotension or volume depletion episode; acute kidney injury.

### 2.4. Study Procedures

Patients were initially evaluated regarding their potential inclusion in the study, according to the prespecified eligibility criteria. Eligible patients provided the investigators with a written informed consent, after a meticulous explanation of the study procedures.

These patients were instructed to visit the Diabetes Center on a scheduled morning after a 12 h period of fasting. A study investigator recorded baseline demographics, anthropometric parameters, medical history and concomitant medication. Blood samples were taken in order to determine glycated hemoglobin, hematocrit, hemoglobin, serum creatinine, estimated glomerular filtration rate, serum sodium, serum uric acid and CRP levels. All patients were instructed to perform a 24 h urine collection ending at the morning of the baseline evaluation to measure urine albumin. Office blood pressure was recorded with a validated oscillometric device and a cuff of appropriate size, with the patient sitting for at least 10 min and with three measurements per occasion taken 2 min apart. Finally, all patients underwent ambulatory blood pressure monitoring (ABPM) with the Mobil-O-Graph^®^ 24 h PWA device (IEM GmbH). Blood pressure monitoring was performed every 20 min during the daytime (7:00 to 23:00) and every 30 min during the nighttime (23:00 to 7:00). Measurements were used for the analysis if >70% of the recordings were valid. Central hemodynamics and arterial stiffness indices were also recorded with the Mobil-O-Graph^®^ 24 h PWA device, as previously described.

The augmentation index was calculated as the augmentation pressure, which is the pressure of the second systolic peak minus the pressure at the inflection point, expressed as the percentage of the pulse pressure and normalized for an HR of 75 bpm (Aix@HR75).

### 2.5. Statistical Analysis

The statistical analysis has been described in detail elsewhere [27]. The change in ambulatory arterial stiffness indices after initiation of SGLT-2 inhibitor treatment is shown below without adjustment for potential confounders (e.g., baseline pharmacotherapy), while we also performed subgroup analyses for the primary efficacy outcome according to the baseline characteristics of interest (e.g., pre-existing cardiovascular disease, treatment with specific drug classes with established beneficial effect on arterial stiffness, etc.). *p*-values lower than 0.05 were considered statistically significant. R software environment has been used for statistical analysis.

## 3. Results

### 3.1. Baseline Characteristics

A total of 46 Caucasian subjects (29 males) were finally enrolled. Due to COVID-19 pandemic restrictive measures imposed by the Greek Ministry of Public Health during the study, the mean follow-up period was extended to 9.98 (3.27) months. A detailed description of participants’ baseline characteristics is provided in a previous publication from this study [27].

In short, the majority of enrolled subjects had concomitant hypertension (69.5%), 60.8% had concomitant dyslipidemia, 56.5% had pre-existing cardiovascular disease, 30.4% of them had background coronary artery disease, 15.2% had a history of cerebrovascular disease and 10.9% had a previous diagnosis of HF. Only 6.5% of the enrolled subjects had previously established CKD.

In terms of baseline antidiabetic treatment, 93.5% of enrolled patients were prior prescribed metformin, while 39.1% were on dipeptidyl-peptidase 4 (DPP-4) inhibitors and one-third (32.6%) on GLP-1RAs treatment. Furthermore, 13.04% of the enrolled patients utilized sulphonylureas, 28.2% of recruited patients were prescribed insulin and only 2.2% were on prior treatment with pioglitazone. Other background pharmacotherapy of interest has been described elsewhere [27].

Of note, patients allocated to empagliflozin did not differ from patients allocated to dapagliflozin across baseline characteristics, except for the use of sulphonylureas (online supplementary Table S1).

### 3.2. Effect of SGLT-2 Inhibitors on Prespecified Outcomes

In Table 1, we present the main study results. From the enrolled participants, 30 were administered dapagliflozin and 16 were administered empagliflozin, according to the treating physician’s clinical discretion. The SGLT-2 inhibitor was associated with an improvement in glycemic control, body weight and BMI, serum uric acid and CRP levels, 24 h urinary protein excretion, office BP and HR; however, none of the observed results reached statistical significance. Of note, the SGLT-2 inhibitor treatment produced a significant increase in hematocrit and hemoglobin levels (*p* < 0.0001 for both). In addition, the SGLT-2 inhibitor treatment was associated with a non-significant increase in TC levels and LDL-C levels. Notably, the SGLT-2 inhibitor treatment resulted in a significant increase in HDL-C levels by 1.5 mg/dL (*p* = 0.04).

**Figure 1:**
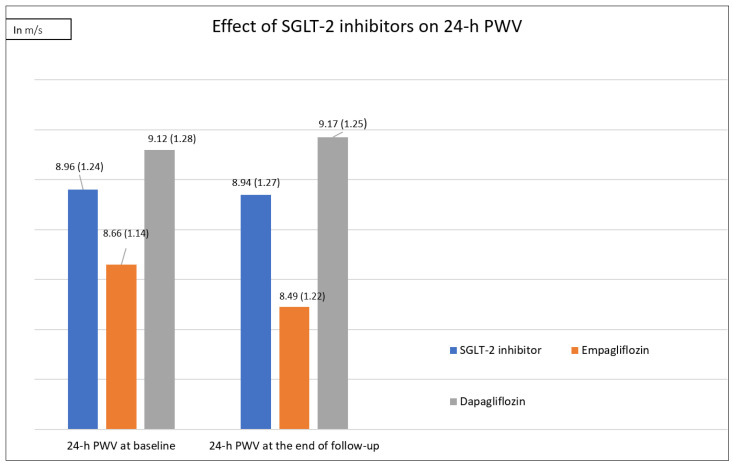
Effect of SGLT-2 inhibitors on 24 h PWV.

Regarding the primary efficacy outcome, we demonstrated that the SGLT-2 inhibitor treatment resulted in a non-significant decrease in PWV by 0.022 m/s (*p* = 0.65), a decrease in daytime PWV by 0.03 m/s (*p* = 0.7) and an increase in nighttime PWV by 0.03 m/s (*p* = 0.33) (Figure 1).

Concerning other indices of arterial stiffness, we showed that the SGLT-2 inhibitor treatment improved Aix and cPP; however, none of the results reached statistical significance (*p* = 0.99 for both outcomes). Regarding central hemodynamics parameters, the SGLT-2 inhibitor treatment improved both cSBP and cDBP; however, both results were non-significant, as well (*p* = 0.80 and 0.90, respectively).

As far as ABPM is concerned, the SGLT-2 inhibitor produced a non-significant decrease in both 24 h SBP and 24 h DBP (*p* = 0.99 for both outcomes). A further analysis revealed that SGLT-2 inhibitors decreased both daytime SBP and DBP (*p* = 0.99 for both outcomes), while they also decreased both nighttime SBP and DBP (*p* = 0.91 and 0.90, respectively).

### 3.3. Effect of Empagliflozin and Dapagliflozin on Arterial Stiffness Indices

An analysis of the derived results per treatment revealed that empagliflozin resulted in a non-significant decrease in PWV (Δ = −0.16 m/s, *p* = 0.93), in daytime PWV (Δ = −0.17 m/s, *p* = 0.91) and in nighttime PWV (Δ = −0.14 m/s, *p* = 0.92), while it produced a non-significant decrease in cPP (Δ = −4 mm Hg, *p* = 0.97) and a non-significant decrease in Aix (Δ = −0.99%, *p* = 0.78) (Figure 1). On the other hand, dapagliflozin resulted in a non-significant increase in PWV (Δ = 0.05 m/s, *p* = 0.19), in daytime PWV (Δ = 0.04 m/s, *p* = 0.24) and in nighttime PWV (Δ = 0.12 m/s, *p* = 0.07) (Figure 1). In addition, dapagliflozin led to a non-significant decrease in cPP levels (Δ = −1.57, *p* = 0.9) and in Aix (Δ = −1.83%, *p* = 0.99). The change in ambulatory arterial stiffness indices with empagliflozin compared to the corresponding change with dapagliflozin did not significantly differ (online supplementary Table S2).

Sub-analyses according to the baseline CVD did not reveal a significant effect either of empagliflozin or dapagliflozin on ambulatory PWV. In addition, prior treatment with drug classes with a beneficial effect on arterial stiffness [namely, renin-angiotensin-aldosterone system (RAAS) blockers and statins] did not have a significant effect on the observed results both for empagliflozin and dapagliflozin.

### 3.4. Correlation of PWV with Other Parameters

We finally assessed the correlation between PWV with other established cardiovascular risk factors. We demonstrated that PWV significantly correlates with body weight (r = 0.38, *p* = 0.008), office SBP (r = 0.47, *p* = 0.001), office DBP (r = 0.47, r = 0.001), 24 h SBP (r = 0.75, *p* < 0.001), 24 h DBP (r = 0.6, *p* < 0.001), cSBP (r = 0.78, *p* < 0.001), cDBP (r = 0.63, *p* < 0.001) and BMI (r = 0.3, *p* = 0.04). No correlation between the change in PWV and the change in glycemic control indices (FPG, HbA1c), in lipid profile parameters or in hematocrit/hemoglobin was demonstrated in our cohort.

## 4. Discussion

This is the first, real-world study available in the literature addressing the effect of two different SGLT-2 inhibitors on ambulatory PWV and other indices of arterial stiffness over a long follow-up period. We have shown that the SGLT-2 inhibitor treatment over almost 1 year of follow-up in a cohort of overweight or obese patients with long-standing T2DM, sub-optimal glycemic control and cardiovascular co-morbidities did not have a significant effect on any of the assessed indices of ambulatory arterial stiffness.

Some of us have previously critically reviewed the studies assessing the effect of SGLT-2 inhibitors on arterial stiffness, reaching the conclusion that the current evidence is limited to provide definite answers, despite the fact that most of the studies demonstrated a beneficial effect of this drug class on PWV [25]. More specifically, a 6-week treatment with empagliflozin was shown to significantly decrease ambulatory PWV compared to the placebo [28]. In another, smaller, randomized controlled trial, a 6-month treatment with canagliflozin compared to perindopril led to a significant decrease in office PWV [29]. Recently, in the largest available randomized controlled trial, researchers found that dapagliflozin compared to placebo over a 3-month period of treatment significantly decreased ambulatory PWV [30]. In another relevant trial, recently published, it was demonstrated that 12-month treatment with empagliflozin resulted in a significant decrease in office PWV, while the effect was greater when combined with liraglutide [31]. In a multicenter, observational study from Japan, researchers found that 12-week treatment with luseogliflozin failed to significantly decrease office PWV [32], generating doubts if there is a drug- and not a class-effect in terms of PWV reduction with SGLT-2 inhibitors [33]. Three observational studies utilizing empagliflozin and dapagliflozin in individuals with T2DM showed a significant reduction in ambulatory or office PWV in 3, 6 and 12 months after initiation [34,35,36].

Other trialists have previously found that 3- and 6-month treatment with empagliflozin led to a significant reduction in cPP [37], while others failed to demonstrate a significant effect of a shorter, 6-week treatment with dapagliflozin on cPP [38]. In addition, another trial showed that a 12-week treatment with dapagliflozin compared to gliclazide provided a significant improvement in Aix [39].

Based on the recent cardiovascular outcome trials proposing that SGLT-2 inhibitors might be crucial for the treatment of special patient sub-populations, such as those with concomitant HF with reduced or preserved ejection fraction [21,22,23] or CKD [20], regardless of baseline T2DM, it seems that there is increasing interest in the underlying mechanisms mediating those beneficial effects. However, according to the current contradictory evidence, the interplay between SGLT-2 inhibitors and arterial stiffness indices does not appear to be catalytic for the mediated cardio- and reno-protection, despite the fact that arterial stiffness is of great importance for the prediction of MACEs, cardiovascular death [9,10] and renal function decline [40].

We found a neutral effect of the SGLT-2 inhibitor treatment on ambulatory PWV in patients with T2DM and either established CVD or certain cardiovascular risk factors. Comparing with two previous trials evaluating the effect of empagliflozin [28] and dapagliflozin [30] on ambulatory PWV, our participants’ baseline characteristics did not differ significantly. However, both studies evaluated the change in ambulatory PWV in 6 and 12 weeks, respectively, while we quantified the corresponding effect of empagliflozin or dapagliflozin after a mean follow-up period of 10 months. Thus, it may be deduced that the numerically small effect of SGLT-2 inhibitors on arterial stiffness can be lost over time.

We have to admit the presence of certain limitations. First, it is a preliminary study with an observational design and a small sample size. The number of enrolled participants is low to reach statistical significance and to draw certain conclusions. Second, we did not evaluate the effect of the SGLT-2 inhibitor on arterial stiffness parameters at shorter time intervals, in order to assess whether there was a short-term, beneficial effect. Third, the absence of a control group to assess the comparative effectiveness of SGLT-2 inhibitors on ambulatory arterial stiffness indices represents an additional limitation. Fourth, the present study took place within the COVID-19 pandemic, with an important adverse impact on patients’ quality of life, physical activity and access to national healthcare services [41]. In addition, we should acknowledge the potential confounding bias in the interpretation of a change in PWV, due to the high prevalence of co-morbidities such as hypertension and dyslipidemia in our cohort, and the beneficial effect of certain drug classes prior prescribed to those subjects (e.g., RAAS blockers, statins, etc.) on arterial stiffness [42]. Finally, our results cannot be generalized due to the single ethnicity nature of the study population.

## 5. Conclusions

In conclusion, we have shown in this real-world study that an SGLT-2 inhibitor treatment did not improve ambulatory arterial stiffness measures over 10 months of follow-up. Based on the limited literature data regarding the effect of SGLT-2 inhibitors on ambulatory arterial stiffness, the promising role of ambulatory arterial stiffness in cardiovascular risk prediction or cardiovascular disease stratification and the established cardio-protective effect of this drug class, it seems that more data are needed. Large, well-designed, prospective studies, ideally randomized controlled trials, are required to shed further light on whether SGLT-2 inhibitors exert any effect on ambulatory arterial stiffness.

## Figures and Tables

**Table 1:** Effect of SGLT-2 inhibitors on major prespecified outcomes.

Parameter of Interest	Baseline	Follow-Up	Change (Δ)	*p*-Value
**PWV (m/s)**	8.96 ± 1.24	8.94 ± 1.27	−0.02	0.65
**Daytime PWV (m/s)**	9.01 ± 1.25	8.98 ± 1.27	−0.03	0.7
**Nighttime PWV (m/s)**	8.83 ± 1.22	8.86 ± 1.29	0.03	0.33
**PWV (m/s)—EMPA**	8.66 ± 1.14	8.49 ± 1.22	−0.16	0.93
**Daytime PWV (m/s)—EMPA**	8.68 ± 1.15	8.51 ± 1.2	−0.17	0.91
**Nighttime PWV (m/s)—EMPA**	8.58 ± 1.1	8.44 ± 1.32	−0.14	0.92
**PWV (m/s)—DAPA**	9.12 ± 1.28	9.17 ± 1.25	0.05	0.19
**Daytime PWV (m/s)—DAPA**	9.18 ± 1.29	9.22 ± 1.25	0.04	0.24
**Nighttime PWV (m/s)—DAPA**	8.96 ± 1.27	9.08 ± 1.24	0.12	0.07
**AIx (%)**	26.22 ± 5.33	24.74 ± 5.07	−1.48	0.99
**AIx (%)—EMPA**	26.13 ± 5.95	25.31 ± 4.62	−0.99	0.78
**AIx (%)—DAPA**	26.26 ± 5.06	24.43 ± 5.34	−1.83	0.99
**c PP (mm Hg)**	50.3 ± 9.56	47.85 ± 8.53	−2.99	0.99
**c PP (mm Hg)—EMPA**	50.5 ± 9.58	46.38 ± 6.31	−4	0.97
**c PP (mm Hg)—DAPA**	50.2 ± 9.72	48.63 ± 9.51	−1.57	0.9
**cSBP (mm Hg)**	115.5 ± 12.91	111.5 ± 11.37	−3.99	0.99
**cDBP (mm Hg)**	78.3 ± 8.96	75.78 ± 7.6	−2.52	0.99
**cSBP (mm Hg)—EMPA**	117.19 ± 13.78	111.63 ± 7.49	−5.56	0.99
**cDBP (mm Hg)—EMPA**	80.25 ± 8.64	76.94 ± 6.06	−3.31	0.99
**cSBP (mm Hg)—DAPA**	114.83 ± 12.58	112.63 ± 13.09	−2.99	0.92
**cDBP (mm Hg)—DAPA**	77.27 ± 9.1	75.17 ± 8.33	−2.1	0.97

Data are presented as mean ± standard deviation, unless otherwise stated. Abbreviations: PWV: pulse wave velocity, EMPA: empagliflozin, DAPA: dapagliflozin, cSBP: central systolic blood pressure, cDBP: central diastolic blood pressure, cPP: central pulse pressure, Aix: augmentation index.

## Supplementary Materials

The following supporting information can be downloaded at: https://www.mdpi.com/article/10.3390/medicina58091167/s1, Table S1: Differences between empagliflozin and dapagliflozin allocated patients across baseline characteristics; Table S2: Change in ambulatory arterial stiffness indices with empagliflozin compared to dapagliflozin.
