# Supplementary material for: Effect of Empagliflozin and Dapagliflozin on Ambulatory Arterial Stiffness in Patients with Type 2 Diabetes Mellitus and Cardiovascular Co-Morbidities: A Prospective, Observational Study
Source: Medicina (Kaunas). 2022 Aug 27;58(9):1167. doi: 10.3390/medicina58091167 (PMC9501055; doi:10.3390/medicina58091167)
Supplement: Supplementary file 1 [file medicina-58-01167-s001.zip › Online supplementary table S1.pdf]

Online supplementary Table S1 Differences between empagliflozin and dapagliflozin allocated patients across baseline characteristics.

Baseline Characteristic	Empagliflozin	Dapagliflozin	Empagliflozin vs. dapagliflozin p value
**Age (years)**	60.25 ± 8.83	64.3 ± 8.16	0.58
**Type 2 diabetes duration (years)**	9.18 ± 6.28	10 ± 6.5	0.65
**Body mass index (kg/m^2)**	32.73 ± 5.52	32.6 ± 5.89	0.69
**Glycated hemoglobin (%)**	7.69 ± 1.77	7.36 ± 1.37	0.65
**Cardiovascular disease**	9 (56.25%)	17 (56.67%)	0.17
**Hypertension**	12 (75%)	20 (66.67%)	0.61
**Coronary artery disease**	5 (31.25%)	9 (30%)	0.35
**Heart failure**	1 (6.25%)	4 (13.33%)	0.12
**Cerebrovascular disease**	2 (12.5%)	5 (16.67%)	0.99
**Dyslipidemia**	12 (75%)	16 (53.33%)	0.37
**Metformin**	15 (93.75%)	28 (93.33%)	0.52
**Insulin**	6 (37.5%)	7 (23.33%)	0.29
**GLP-1 receptor agonists**	7 (43.75%)	8 (26.67%)	0.99
**DPP-4 inhibitors**	6 (37.5%)	12 (40%)	0.64
**Sulphonylureas**	3 (18.75%)	3 (10%)	0.009
**RAAS blockers**	12 (75%)	17 (56.67%)	0.61
**Calcium channel blockers**	5 (31.25%)	8 (26.67%)	0.99
**Diuretics**	4 (25%)	14 (46.67%)	0.99
**B-blockers**	7 (43.75%)	11 (36.67%)	0.64
**Antiplatelet agents**	11 (68.75%)	13 (43.33%)	0.33

**Statins**	14 (87.5%)	17 (56.67%)	0.052
