# Supplementary material for: Effect of Empagliflozin and Dapagliflozin on Ambulatory Arterial Stiffness in Patients with Type 2 Diabetes Mellitus and Cardiovascular Co-Morbidities: A Prospective, Observational Study
Source: Medicina (Kaunas). 2022 Aug 27;58(9):1167. doi: 10.3390/medicina58091167 (PMC9501055; doi:10.3390/medicina58091167)
Supplement: Supplementary file 1 [file medicina-58-01167-s001.zip › Online supplementary table S2.pdf]

<u>Online supplementary Table S2</u> Change in ambulatory arterial stiffness indices with empagliflozin compared to dapagliflozin.

**Change in arterial stiffness parameter**	**Empagliflozin vs. dapagliflozin**	**p-value**
**Δ 24-h PWV (m/s)**	-0.085	0.19
**Δ daytime PWV (m/s)**	-0.07	0.27
**Δ nighttime PWV (m/s)**	-0.085	0.09
**Δ Aix (%)**	0.57	0.074
**Δ cPP (mm Hg)**	-4.71	0.26
